# Evaluation of a rollout of alcohol brief interventions in health- and social-care teams following multidisciplinary training

**DOI:** 10.1186/1940-0640-7-S1-A84

**Published:** 2012-10-09

**Authors:** Niamh Fitzgerald, Heather Molloy, Fiona MacDonald

**Affiliations:** 1 Create Consultancy, Ltd., and Robert Gordon University, Aberdeen, Scotland, UK; 2 East Renfrewshire Community Health and Care Partnership, East Renfrewshire, UK

## 

There is a robust body of evidence supporting alcohol brief intervention (BI) in primary health-care settings, but fewer studies have explored delivery elsewhere. This qualitative evaluation followed up staff in one integrated health and social-care service in Scotland to find out if and how multidisciplinary training in alcohol BI impacted practice, and if and why BI had been delivered following the training. Nineteen semi-structured in-depth qualitative telephone interviews were carried out among 10 of the 89 practitioners who had attended the course as well as among managers and administrators from various teams (e.g., social work, elder care, and community mental health). Interviews were recorded, transcribed, and analyzed thematically. All quotations were checked with interviewees. Participants felt that training had improved their knowledge and confidence around alcohol and were supportive of having a role in delivering BI, but very few had actually delivered any. Practitioners perceived that their clients did not need alcohol BI for a variety of reasons, including that they either drank too much or too little to merit one. Despite this, practitioners described giving advice on alcohol but failed to recognize these conversations as opportunities to deliver BIs. A range of other barriers to delivery emerged, including the view that specific screening, delivery techniques, and monitoring of BIs did not fit with their current practice and assessment procedures, which already included (sometimes unhelpful) questions or questionnaires on alcohol use. The barriers to delivery were at individual, team, and service levels and are likely to be best addressed by a strategic approach of which training is only one part. The findings also suggest the need to take a setting-specific approach to efforts to embed BI delivery into routine practice.

